# Keratitis-Ichthyosis-Deafness Syndrome, Atypical Connexin GJB2 Gene Mutation, and Peripheral T-Cell Lymphoma: More Than a Random Association?

**DOI:** 10.1155/2011/848461

**Published:** 2011-08-10

**Authors:** Claudio Fozza, Fausto Poddie, Salvatore Contini, Antonio Galleu, Francesca Cottoni, Maurizio Longinotti, Francesco Cucca

**Affiliations:** ^1^Institute of Hematology, University of Sassari, 07100 Sassari, Italy; ^2^Institute of Medical Genetics, University of Sassari, 07100 Sassari, Italy; ^3^Institute of Dermatology, University of Sassari, 07100 Sassari, Italy

## Abstract

Keratitis-ichthyosis-deafness (KID) syndrome is a rare congenital disorder characterized by skin lesions, neurosensorial hypoacusia, and keratitis, usually due to the c.148G → A mutation involving the connexin 26 gene. We report on a KID patient who showed the atypical c.101T → C mutation and developed a T-cell lymphoma so far never described in this group of patients.

## 1. Introduction

Keratitis-ichthyosis-deafness (KID) syndrome is a rare congenital disorder characterized by a variety of skin lesions—that is, palmoplantar keratoderma, thickening of the skin, and erythematous verrucous lesions—neurosensorial hypoacusia, and keratitis with a variable degree of visual impairment [1]. Both sporadic and familial forms of the syndrome have been described, the latter usually showing a dominant pattern of inheritance [2]. The molecular lesion responsible for the syndrome typically involves the connexin 26 (Cx26) gene (GJB2). Most patients display the heterozygous c.148G→A mutation causing the substitution of an aspartic acid for an asparagine at position 50 (p.Asp50Asn), while a few of them show the c.50C→T mutation, implying the substitution of a serine for a phenylalanine at position 17 (p.Ser17Phe) [2]. However, even a mutation in the connexin 30 (Cx30) gene (GJB6) has been found in a typical KID patient [3], thus suggesting a genetic heterogeneity of the syndrome. As connexins are a large family of small integral membrane proteins which influence tissue cornification by modulating the establishment of direct cell-cell communication through gap junction channels [4], it is likely that defects involving this class of proteins are at the basis of the well-known increased incidence of squamous cell carcinoma in KID patients [5]. 

## 2. Case Presentation

Here we report on an adult patient with a typical KID syndrome who developed a peripheral T-cell lymphoma. It is worth noting that sequencing of GJB2 and GJB6 genes revealed only a Cx26 (GJB2) c.101T→C mutation, a variant usually associated with isolated hearing impairment [6, 7]. 

**Figure 1:**
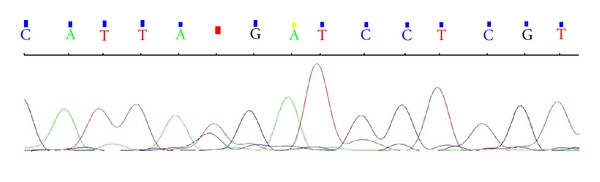
A search for mutations within the connexin 26 gene GJB2 showed the heterozygous c.101T→C mutation (in red in the figure) causing the substitution of a methionine residue for threonine at position 34 (p.Met34Thr).

Briefly, the patient presented skin ichthyosis since his adolescence and in subsequent years developed severe bilateral hypoacusia and keratitis. The coexistence of such progressively worsening features pointed to the clinical diagnosis of KID syndrome. At that time, no molecular investigations were performed. The patient came to our attention in November 2007, when he was 65 years old, with diffuse lymphoadenopathy and splenomegaly (122 mm) associated to thrombocytopenia (84 × 10^9^/L), neutropenia (1.4 × 10^9^/L), and elevated lactate dehydrogenase level (1578 U/L) along with a worsening of his erythematosus desquamating cutaneous rash. After an inguinal node biopsy, a diagnosis of CD3+, CD45RO+, bcl2+, and CD7+ peripheral T-cell non-Hodgkin lymphoma (NHL) was made. Because of bone marrow involvement in trephine biopsy, the lymphoma stage resulted to be IV A with a high risk on the International Prognostic Index (IPI). Besides an infiltration by T-lymphoma cells, the skin biopsy showed epidermal cysts, hyperkeratotic lesions, and inflammatory nodules. The ophttalmoscopic and audiometric evaluations showed bilateral neurosensorial hypoacusia and superficial punctate keratitis. All these findings being compatible with a fully expressed KID phenotype, the GJB2 gene sequencing was firstly performed. Briefly, after genomic DNA extraction from peripheral blood following the standard salting-out procedures, GJB2 was amplified by PCR using the primers reported in Table 1. PCR products were then sequenced on an ABI Prism 3130 genetic analyzer by using BigDye Terminator v3.1 (Applied Biosystems) showing the heterozygous c.101T→C mutation (Figure 1), which causes the substitution of a methionine residue for threonine at position 34 (p.Met34Thr, briefly M34T). Both the GJB2 c.148G→A and c.50C→T gene mutations usually found to be associated with KID syndrome [2] were excluded. The sequencing analysis was then extended to the Cx30 GJB6 coding gene but failed to reveal any further mutation. 

Our patient was treated with a combination of chemotherapy including Cyclophosphamide, Doxorubicin, Vincristine, and Prednisone and immunotherapy with Alemtuzumab. After a partial response, the patient died of *Cytomegalovirus* pneumonia 7 months after the diagnosis of T-cell lymphoma.

## 3. Discussion

The present case deserves some comments. Firstly, the M34T mutation causing the substitution of a methionine residue for threonine at position 34 (p.Met34Thr) has never been described in patients with typical KID syndrome, whereas it has already been found in a homozygous as well as in a double heterozygous state in subjects with isolated hearing impairment. However, even in these cohorts this mutation was reported with extremely low frequencies [6, 7]. In addition, as the M34T variant has an allele frequency of about 1% even the in the whole European healthy population [8], we ought to conclude that the pathogenetic role of the M34T variant in our KID patient has still to be proved. Secondly, an increased susceptibility to cutaneous cancer has been reported in subjects with KID syndrome [5]. Considering that the CX26 gene modulates the cadherin expression [9], it is probable that such a susceptibility may be related to the cadherin downregulation described in approximately 70% of squamous cell carcinoma patients [10]. On the other hand T-cell NHLs are rare malignancies accounting for 10% to 15% of all NHLs [11]. Cadherin is expressed and functionally active even in T-lymphoma cells, implying a possible involvement in the mechanisms of lymphoma cell dissemination to skin and central nervous system [12]. Therefore, the coexistence of KID syndrome and T-cell lymphoma may be more than a coincidence. In the same way as the gene sequencing of GJB2 and GJB6, with the exception of the M34T variant, did not reveal any of the molecular defects typical of KID syndrome, we are tempted to conclude that such an association of three extremely rare conditions in the same patient might not be merely accidental.

## Figures and Tables

**Table 1:** GJB2 forward and reverse primers.

GJB2 FW F1	CATTCGTCTTTTCCAGAGCA

GJB2 RV F1	CACGTGCATGGCCACTAG

GJB2 FW F2	CGTGTGCTACGATCACTAC

GJB2 RV F2	AGCCTTCGATGCGGACCTT

GJB2 FW F3	ACCGGAGACATGAGAAGAAG

GJB2 RV F3	TTCCAGACACTGCAATCATG

GJB2 FW F4	TATGTCATGTACGACGGCT

GJB2 RV F4	TCTAACAACTGGGCAATGC

## References

[1] Skinner BA, Greist MC, Norins AL (1981). The keratitis, ichthyosis, and deafness (KID) syndrome. *Archives of Dermatology*.

[2] Mazereeuw-Hautier J, Bitoun E, Chevrant-Breton J (2007). Keratitis-ichthyosis-deafness syndrome: disease expression and spectrum of connexin 26 (GJB2) mutations in 14 patients. *British Journal of Dermatology*.

[3] Jan AY, Amin S, Ratajczak P (2004). Genetic heterogeneity of KID syndrome: identification of a Cx30 gene (GJB6) mutation in a patient with KID syndrome and congenital atrichia. *Journal of Investigative Dermatology*.

[4] Richard G (2000). Connexins: a connection with the skin. *Experimental Dermatology*.

[5] Grob JJ, Breton A, Bonafe JL (1987). Keratitis, ichthyosis and deafness (KID) syndrome. Vertical transmission and death from multiple squamous cell carcinomas. *Archives of Dermatology*.

[6] Azaiez H, Chamberlin GP, Fischer SM (2004). GJB2: the spectrum of deafness-causing allele variants and their phenotype. *Human Mutation*.

[7] Batissoco AC, Abreu-Silva RS, Braga MC (2009). Prevalence of GJB2 (connexin-26) and GJB6 (connexin-30) mutations in a cohort of 300 Brazilian hearing-impaired individuals: implications for diagnosis and genetic counseling. *Ear and Hearing*.

[8] Feldmann D, Denoyelle F, Loundon N (2004). Clinical evidence of the nonpathogenic nature of the M34T variant in the connexin 26 gene. *European Journal of Human Genetics*.

[9] Stoler AB, Stenback F, Balmain A (1993). The conversion of mouse skin squamous cell carcinomas to spindle cell carcinomas is a recessive event. *Journal of Cell Biology*.

[10] Koseki S, Aoki T, Ansai S (1999). An immunohistochemical study of E-cadherin expression in human squamous cell carcinoma of the skin: relationship between decreased expression of E- cadherin in the primary lesion and regional lymph node metastasis. *Journal of Dermatology*.

[11] Vose JM (2008). Peripheral T-cell non-Hodgkin’s lymphoma. *Hematology/Oncology Clinics of North America*.

[12] Kawamura-Kodama K, Tsutsui J, Suzuki ST (1999). N-cadherin expressed on malignant T cell lymphoma cells is functional and promotes heterotypic adhesion between the lymphoma cells and mesenchymal cells expressing N-cadherin. *Journal of Investigative Dermatology*.

